# Renal Disease and Systemic Sclerosis: an Update on Scleroderma Renal Crisis

**DOI:** 10.1007/s12016-022-08945-x

**Published:** 2022-06-01

**Authors:** Alice Cole, Voon H. Ong, Christopher P. Denton

**Affiliations:** UCL Centre for Rheumatology and Connective Tissue Diseases, Royal Free Campus, Rowland Hill Street, London, NW3 2PF UK

**Keywords:** Systemic sclerosis, Scleroderma renal crisis, Thrombotic microangiopathy, Complement, Acute kidney injury

## Abstract

Scleroderma renal crisis (SRC) is a life-threatening complication of systemic sclerosis (SSc) with a mortality of 20% at 6 months. Once the leading cause of mortality in scleroderma (SSc), it remains a serious complication, often necessitating level three care for patients affected. Whilst renal outcomes have significantly improved following the advent of angiotensin-converting enzyme inhibitor (ACEi) therapy, SRC remains a precarious challenge for clinicians, due to lack of preventative measures and the fact that patients can rapidly decline despite best medical management. Large cohort studies spanning decades have allowed clear identification of phenotypes particularly at risk of developing SRC thus allowing enhanced monitoring and early identification in those individuals. Novel urinary biomarkers for renal disease in SSc may offer a new window for early identification of SRC patients and response to treatment. Multiple studies have demonstrated increased activity of complement pathways in SRC with some anecdotal cases exhibiting serological response to treatment with eculizumab where ACEi and therapeutic plasma exchange (TPE) were not successful. Endothelin-1 blockade, a therapeutic strategy in other SSc vasculopathies, has shown potential as a target but clinical trials are yet to show a clear treatment benefit. Clear guidelines for the management of SRC are in place to standardise care and facilitate early collaboration between rheumatology and renal physicians. Outcomes following renal transplant have improved but the mortality of SRC remains high, indicating the need for continued exploration of the mechanisms precipitating and exacerbating SRC in order to develop novel therapies.

## Introduction

Scleroderma renal crisis (SRC) is a life-threatening complication of systemic sclerosis (SSc) with a mortality of 20% at 6 months. Once the leading cause of mortality in Scleroderma (SSc), it remains a serious complication, often necessitating level three care for patients affected. Whilst renal outcomes have significantly improved following the advent of angiotensin-converting enzyme inhibitor (ACEi) therapy, SRC remains a precarious challenge for clinicians, due to lack of preventative measures and the fact that patients can rapidly decline despite best medical management. Large cohort studies spanning decades have allowed clear identification of phenotypes particularly at risk of developing SRC thus allowing enhanced monitoring and early identification in those individuals. Emerging data surrounding the pathophysiology of SRC has suggested encouraging targets such as endothelin-1 and upregulated complement pathways which may lead to novel changes in our management of this patient group.

## Overview

The earliest description considered to represent SRC originated from Auspitz in 1863 who described the rapid death of a patient with thickened skin and uraemia [1]. In the late 1930s, some of the histological hallmarks of SRC such as intimal hyperplasia of the renal vessels and fibrinoid degeneration in interlobular arteries were described. In one of these cases, the patient had been diagnosed with SSc and obliterative endarteritis of the kidney [2]. The histological abnormalities observed in the kidneys were also described in SSc patients who did not suffer from SRC [3]. The term ‘renal-crisis’ was coined by Moore and Sheehan in 1952 [4]. Treatment of SRC has historically consisted of aggressive anti-hypertensive therapy using methyldopa or propranolol with dialysis and in some cases bilateral nephrectomy. Mortality significantly reduced with the introduction of ACEi in the 1980s; however, European League Against Rheumatism Scleroderma Trials and Research (EUSTAR) data has shown no other significant impact on mortality in the post-ACE era. The frequency of SRC does seem to be reducing which may be due to the more widespread use of vasodilator therapy to treat complications of SSc such as Raynaud’s phenomenon and pulmonary arterial hypertension (PAH) [5]. It may also be related to the more judicious use of glucocorticoids in patient with SSc due to the recognition that steroids may provoke SRC [6]. Recent studies have also demonstrated that outcome of renal transplant in SRC have improved and are now comparable to other causes of end-stage renal failure (ESRF) [7].

## Classifying Renal Disease in Scleroderma

Separate disease entities exist within the kidney in SSc. SRC classically presents with accelerated hypertension and acute kidney injury (AKI) defined as an increase in serum creatinine > 1.5 × baseline. Whilst SRC should certainly be included in the differential for such a presentation, other causes to consider include anti-neutrophil cytoplasmic antibody (ANCA)–associated vasculitis, membranous nephritis, other primary causes of thrombotic microangiopathies (TMA) such as thrombotic thrombocytopenic purpura (TTP) or disseminated intravascular coagulopathy (DIC). Other non-immune-mediated causes such as renal artery stenosis may mimic the presentation of SRC. There are a proportion of SSc patients who have unexplained renal abnormalities such as proteinuria and the significance of this is not fully recognised.

In 2015, the Scleroderma Clinical Trials Consortium (SCTC) working group conducted a scoping review and a consensus study to produce classification criteria for SRC which could be widely used in research. The group have identified a core set of variables which define SRC, and these are currently being used on real-world patients as part of the International Scleroderma Renal Crisis Survey II (ISRCS II) to validate the set and provide data on specificity. The main parameters outlined are AKI, hypertension, microangiopathic haemolytic anaemia (MAHA) and thrombocytopenia, target organ dysfunction, and renal histopathology (Table 1 [8]).

**Table 1:** Classification criteria for SRC as defined by the Scleroderma Clinical Trials Consortium (SCTC) working group [8]

Domain
**Blood pressure**
Acute increase in blood pressure defined as any of the following:
- Systolic blood pressure ≥ 140 mm Hg
- Diastolic blood pressure ≥ 90 mm Hg
- An increase in systolic blood pressure of ≥ 30 mm Hg above normal
- An increase in diastolic blood pressure of ≥ 20 mm Hg above normal
Blood pressure measurement should be taken twice, separated by at least 5 min; if blood pressure readings are discordant, repeat readings should be taken until 2 consistent readings are obtained
**Kidney injury** [75]
AKI defined as any of the following:
- Increase in serum creatinine of ≥ 26.5 μmoles/l (≥ 0.3 mg/dl) within 48 h
- Increase in serum creatinine to ≥ 1.5 times baseline, which is known or presumed to have occurred within the prior 7 days
- Urine volume < 0.5 ml/kg/h for 6 h
**MAHA and thrombocytopenia**
New or worsening anaemia not due to other causes
Schistocytes or other red blood cell fragments on blood smear
Thrombocytopenia ≤ 100,000 platelets/mm^3^, confirmed by manual smear
Laboratory evidence of haemolysis, including elevated lactate dehydrogenase, reticulocytosis, and/or low or absent haptoglobin
A negative direct antiglobulin test
**Target organ dysfunction**
Hypertensive retinopathy (haemorrhages, hard and soft [cottonwool] exudates, and/or disc oedema, not attributable to other causes), confirmed by an ophthalmologist
Hypertensive encephalopathy, characterized by headache, altered mental status, seizures, visual disturbances, and/or other focal or diffuse neurologic signs not attributable to other causes
Acute heart failure, characterized by typical symptoms (e.g., breathlessness, ankle swelling, and fatigue) that may be accompanied by signs (e.g., elevated jugular venous pressure, pulmonary crackles, and peripheral oedema)
Acute pericarditis, diagnosed with at least 2 of the 4 following criteria: (1) pericarditis chest pain, (2) pericardial rub, (3) new widespread ST segment elevation or PR segment depression on electrocardiography, and (4) pericardial effusion (new or worsening) on cardiac echocardiography
**Renal histopathology**
Histopathologic findings on kidney biopsy consistent with SRC, which may include the following:
- Small vessel (arcuate and interlobular arteries) changes that predominate over glomerular alterations
- Glomerular changes of thrombotic microangiopathy may be present, with acute changes including fibrin thrombi and endothelial swelling, red blood cell fragments, and mesangiolysis, and chronic changes including double contours of the glomerular basement membrane
- Nonspecific ischemic changes with corrugation of the glomerular basement membrane, and even segmental or global sclerosis of glomeruli may occur
- Early vascular abnormalities include intimal accumulation of myxoid material, thrombosis, fibrinoid necrosis, and fragmented red blood cells, sometimes resulting in cortical necrosis
- Narrowing and obliteration of the vascular lumen lead to glomerular ischemia. Juxtaglomerular apparatus hyperplasia, while relatively rare (10%), can be observed
- Late changes are manifested by intimal thickening and proliferation (which lead to characteristic vascular ‘onion-skin’ lesions), glomerulosclerosis, and interstitial fibrosis
- Nonspecific tubular changes may also occur, including acute tubular injury in the early stage of injury, and later interstitial fibrosis and tubular atrophy. Since none of these findings is specific for SRC, the pathologic diagnosis must be supported by appropriate clinical and serologic data

## Epidemiology

**Fig. 1:**
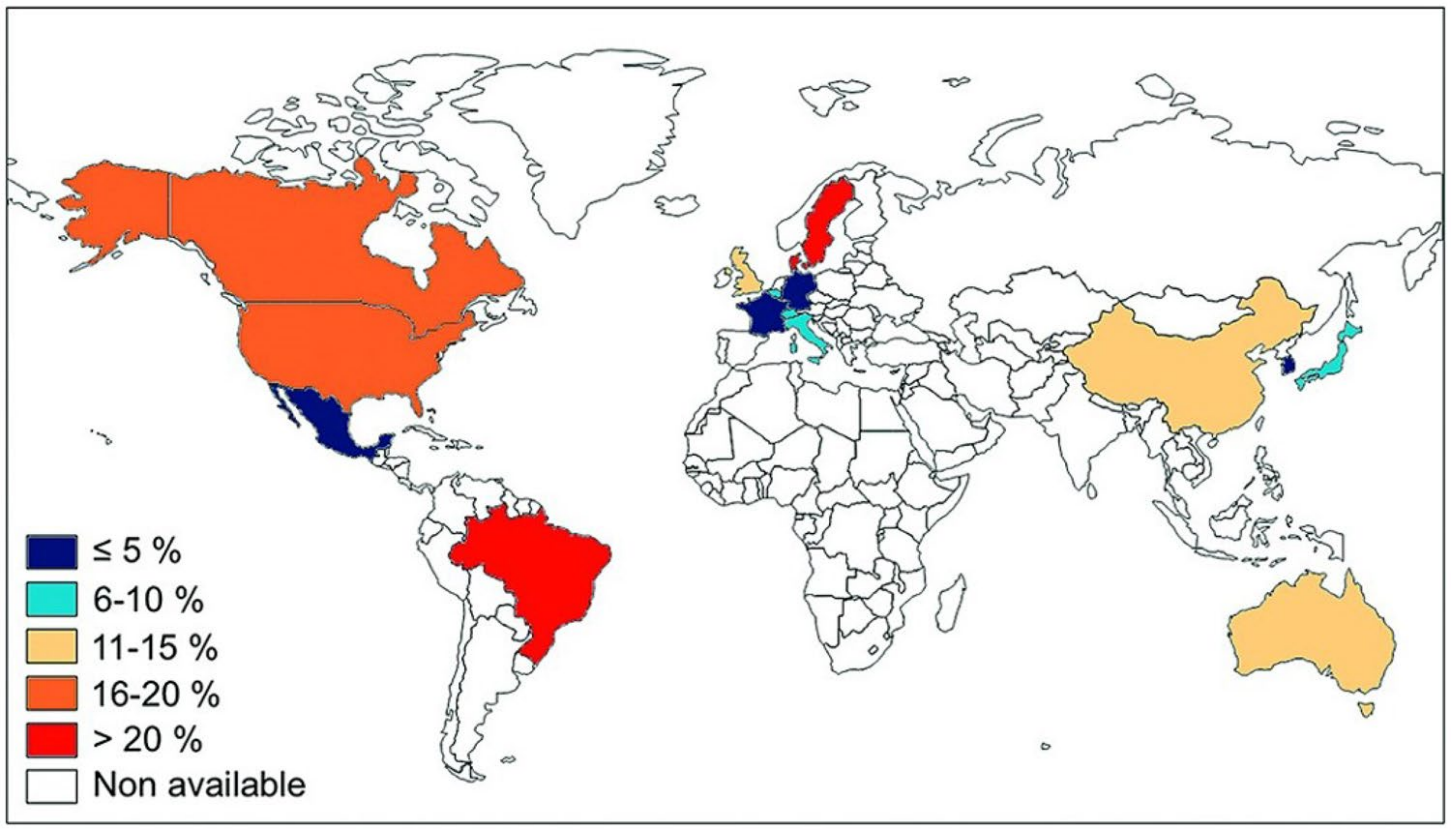
Worldwide prevalence of anti-RNA polymerase III antibody according to French systematic review and meta-analysis. Reproduced with permission from Sobanski et al. [14]

Initial studies reported the prevalence of SRC in early diffuse disease be as high as 25%; however, a 2016 meta-analysis demonstrated that the frequency is now as low as 5% [9], with the US Prospective Registry in Systemic Sclerosis (PRESS) cohort, reporting a 10% frequency [10]. Studies in the United Kingdom (UK) have shown frequency of 14% in diffuse patients, whereas the frequency of SRC amongst limited patient remained low at 3% [11]. Studies carried out in Japan have found the lowest reported frequencies of SRC between 1 and 3% [12, 13]. The highest rates of SRC have been observed in the USA, UK, and Australia [9]. The distribution of SRC between patient populations is likely to link to the heterogeneity between groups and the varying prevalence of autoantibodies associated with SRC such as anti RNA polymerase III, as seen in Fig. 1 [14]. Studies have shown that the highest rates of anti RNA polymerase III have been observed in North America (14%) [14], correlating with SRC prevalence.

The 2016 meta-analysis by Turk et al. reported no significant change in SRC prevalence temporally; however, there was a non-significant (*p* = 0.16) reduction in SRC frequency observed in the diffuse group when analysing cohort by year. This non-significant finding would match with the general expert consensus that cases of SRC do appear to have fallen over time. A possible explanation for this could include the wider awareness of the patients at risk of SRC and the subsequent reduction in use of glucocorticoids and cyclosporine in these patients.

Renal crisis is classically reported to occur in the ‘early’ years of disease (less than 5 years from first non-Raynaud’s symptom), with 75% of cases occurring in years 1 to 4. A large German retrospective analysis found that the distribution of SRC between males and females was representative of the SSc cohort as whole, with a female predominance of 3:1. After univariable analysis, there was no significant difference between sex and risk of SRC (*p* = 0.063) [15]. Clinical risk factors for the development of SRC, including diffuse disease, are discussed in a later section. One published case series found that 22% of SRC represented the patient’s first clinical presentation of the disease, even if other features of disease had preceded the crisis [11].

Late presentation of SRC should not be overlooked. Cases up to 20 years after diagnosis have been reported. It is possible that the presentation of SRC in these cases is affected by immunosuppression received for other complications of SSc, such as skin disease.

An analysis of the Genome Research in African American Scleroderma Patients (GRASP) cohort highlighted the severe disease burden amongst African Americans, demonstrating that the prevalence of SRC is 7%, 3.5 times higher than the 2% prevalence reported after analysis of the EUSTAR cohort [16]. These figures are not adjusted for prevalence of anti-RNA polymerase III antibody as this serological test was only available after 2007 and thus missing for 40% of the GRASP cohort.

## Pathogenesis

**Fig. 2:**
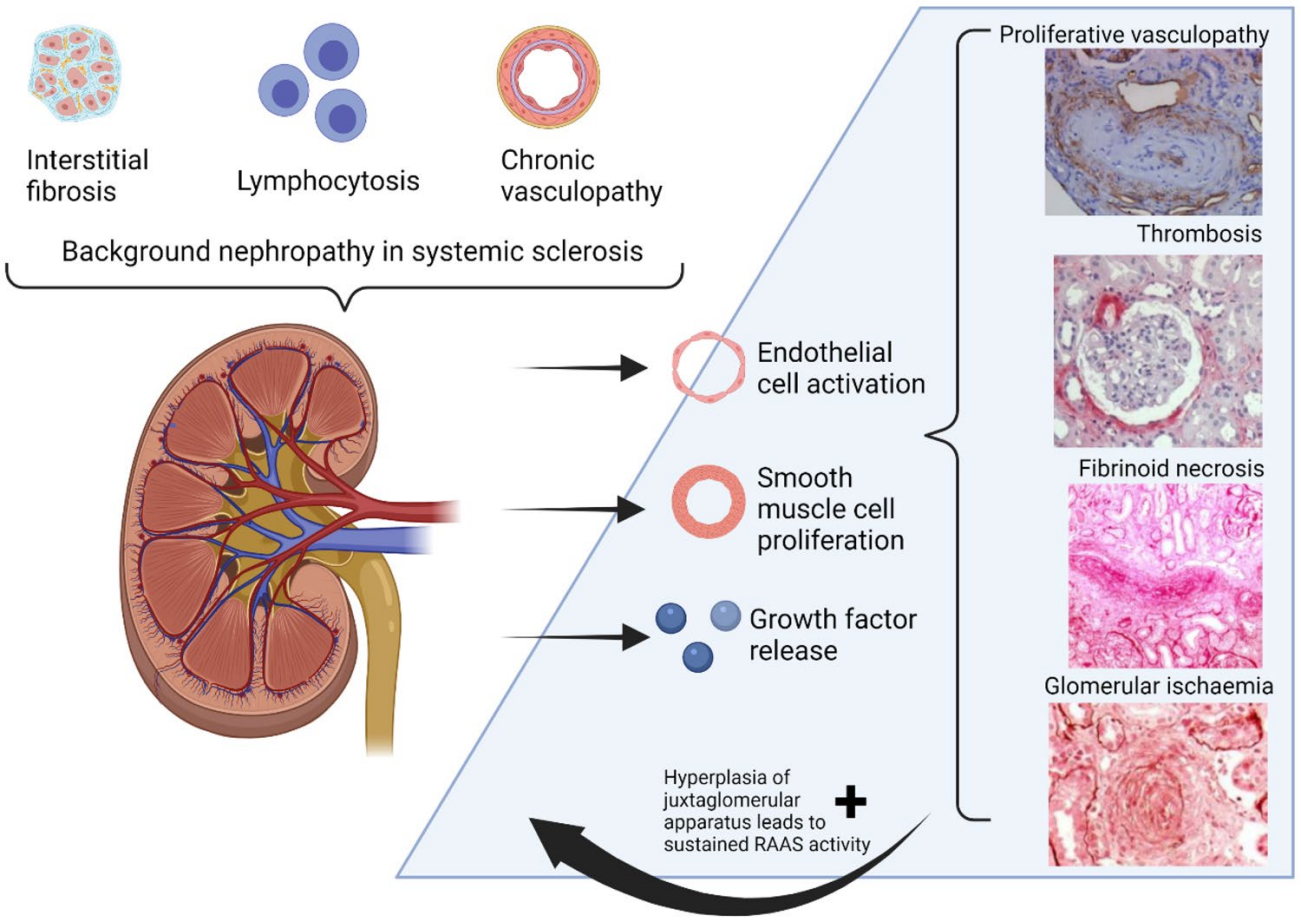
Proposed pathogenesis of SRC. Modified from Denton et al. [18]. Created with BioRender.com

SRC arises from reduced blood supply to the kidney which is proposed to occur in susceptible individuals for a variety of reasons. Susceptible individuals with SSc have the unifying abnormal intra-renal features of vasculopathy, fibrosis, and autoimmunity which allow injury to the vessel wall to initiate an amplification loop of local damage and activation of the renin-aldosterone-angiotensin (RAA) axis and hence SRC, as demonstrated by Fig. 2 [17]. Autopsy specimens have shown that interstitial fibrosis, lymphocytosis, and chronic vasculopathy are often present in SSc without renal crisis but a yet-to-be-defined event, possibly vascular, triggers the endothelial activation and release of growth factors and cytokines which then leads to smooth muscle proliferative vasculopathy. Proliferative vasculopathy leads to glomerular ischaemia and sustained activation of the RAA axis with hyperplasia of the juxtaglomerular axis [18]. The improvement observed after inhibition of the RAA axis with ACEi suggests that hypereninaemia plays a clear role in the pathogenesis of SRC; however, a prospective study showed that increased renin levels are not predictive of subsequent SRC [19], suggesting that other factors are involved. Other factors believe to play a role in reduction of glomerular blood flow include decreased cardiac output due to cardiac scleroderma or heart failure, direct effects of angiotensin II, glucocorticoids, and renal vasospasm, i.e. ‘renal Raynaud’s’ [20].

In 2019, a Japanese group proposed that there is further definition to be made in the pathophysiology of SRC [21]. This group explained that there is clear pathological difference between two groups in SSc, with narrowly defined SSc (nd-SSc) vasculopathy causing intimal thickening and subsequent hypertension whereas SSc-TMA is associated with a lesion in the vessel wall leading to microvascular thrombosis. These pathologies overlap in presentation but do appear to have different disease trajectories; nd-SRC being associated with initial elevated blood pressure and serum creatinine associated with a milder thrombocytopenia later in the disease course. Conversely, SSc-TMA was associated with early and severe thrombocytopenia, followed by elevated blood pressure and creatine. In this study SSc-TMA was associated with steroids. The working group suggested we differentiate between the subtypes clinically by the sequence of thrombocytopenia, elevated BP, and elevated creatinine where possible. This is potentially more helpful than the current grouping of ‘hypertensive’ and ‘normotensive’ as it relates to mechanism of injury. However, if differentiation relies on histology via renal biopsy in the acutely unwell patient, this may produce a practical barrier to the uptake of this classification system.

### Renal Biopsy Abnormalities

The overall pathological picture can be characterized by endothelial damage and thrombus formation. Unlike atypical haemolytic uraemic syndrome (aHUS), small vessel thrombus are more prevalent than glomerular thrombus [22]. SRC demonstrates predominant small vessel involvement with early changes such as mucoid intimal oedema, thrombosis, and fibrinoid necrosis with later intimal thickening leading to obliteration of the lumen which gives an ‘onion skin’ appearance under microscopy. Work is currently ongoing to define the characteristic renal biopsy abnormalities observed in SRC as part of the ISRCC II study [8]. Adventitial and peri-adventitial fibrosis is also observed which indicates a chronic vasculopathy process. Interestingly the extent of fibrosis does not reflect long-term renal outcome [23]. In extensive histological studies of SRC, it has been found that the extent of acute vascular injury, glomerular ischaemic collapse, and C4d deposits are linked to delayed recovery or failure to recover renal function.

Despite biopsy findings providing good prognostic information, they are not routinely used in the acute setting as they are often not required to confirm diagnosis and the invasive procedure carries significant risk in the setting of hypertension and thrombocytopenia, so is reserved for cases where other diagnoses are being considered.

### Complement

There is evidence that cases of SSc-TMA with normal ADAMTS13 levels (i.e. not TTP) have responded well to TPE, albeit alongside treatment with ACEi. This response may suggest that SSc-TMA is mediated by an unrestricted complement cascade, which could occur due to genetic mutations or autoantibodies against complement regulator proteins as seen in aHUS [24]. A 2012 analysis of the EUSTAR database found 5.2% prevalence of hyocomplementaemia (defined as low C3 or C4 levels) in SSc patients but using multivariate analysis, this was not associated with any specific disease parameter such as SRC [25].

Further evidence of complement activity was found in a Spanish case series of 29 TMA patients, where immunofluorescence showed increased deposition of C5b-9 in the endothelium of renal arterioles and in glomeruli [26]. Serum samples from the patients induced C5b-9 ex vivo and demonstrated increased soluble C5b-9 activity. Serum ratios of complement factor 3-d (C3d): complement factor 3 (C3) and Factor B: Factor Bb (FB:FBb) were also increased, which would agree with the hypothesis of increased alternate pathway activity. It is important to note, whilst revealing some important insights with regard to the role of complement in TMA, the 2 cases of SRC in this series did not respond to treatment with eculizumab.

There have however been multiple anecdotal cases of successful response to eculizumab, the monoclonal antibody directed against complement factor 5 (C5) which blocks production of C5b-9 and therefore formation of the membrane attack complex (MAC) complex [27, 28]. Cases treated with eculizumab had evidence of MAHA and showed a dramatic improvement in renal function following treatment but mortality was high due to the severity of SRC observed in these cases [27, 29, 30]. Genetic screening in these cases did not reveal any genetic mutations for the complement proteins or their regulators which has been observed in aHUS.

Whilst there is a growing body of evidence that aberrant complement activation is involved in SRC, we are yet to identify the specific mechanisms which result in the vascular structural abnormalities observed or trigger such changes, raising the question of cause or effect with regard to complement in SRC. However, based on the positive anecdotal evidence, it is reasonable to treat SRC patients with eculizumab in whom complement mediated TMA is suspected, who have not responded to ACEi or TPE.

### Endothelin

The endothelin axis has a recognised role in SSc vasculopathy of digital ulceration and PAH. Studies directed at SRC showed increased levels of endothelin-1 and increased expression of endothelin A and B receptors in SRC [31–33]. Endothelin receptor antagonist have been explored in SRC; however, results with bosentan, a selective endothelin-1 receptor antagonist, did not improve renal outcome in SRC [34]. The preliminary report of a phase II randomised controlled trial of zibotentan, an endothelin-A antagonist, in SSc-CKD did find a statistical change in urinary MCP-1 and stabilisation of estimated glomerular filtration rate (eGFR) [35].

### Biomarkers

Adipose tissue–derived cytokines (adipokines) are thought to be important mediators of immunity. Liopcalin-2 levels were measured in treatment-naïve SSc patients and levels were significantly raised in cases of SRC (*n* = 2) and showed positive correlation with modified Rodnan skin score (mRSS) [36].

Endothelial damage is associated with expression of adhesion molecules such as soluble vascular cell adhesion molecule (sVCAM) which have previously been shown to correlate with disease severity and in the highest recorded case in one particular study, did precede a case of SRC [37].

Serum soluble CD147 has also been investigated to determine whether it has a role in SRC pathogenesis. One study found that despite there not being a difference in CD147 levels between limited and diffuse patients, higher levels of CD147 were associated with SRC (0.13 SSc, 0.0 control, *p* < 0.05) [38].

### Animal Models of SRC

There are not yet any established animal models of SRC. The TβRIIΔk-fib transgenic mouse model replicates hypertension and large vessel fibrosis. The model demonstrates exaggerated fibrotic response to hypertensive injury and provides opportunity for further studies into the specific mechanism of injury in SRC [39].

### Emerging

Studies have been carried out to determine whether SSc-specific autoantibodies not only stratify patient groups but also play an active role in the pathophysiology of SSc. There is evidence of autoimmunity towards AT(1)R and ET(A)R receptors on endothelial cells which increase TGF-beta expression [40].

## Risk Factors

### Scleroderma-Specific Antibodies

It has been widely described that the phenotype most at risk for development of SRC are those patients with early diffuse SSc with proximal skin thickening [16, 41]. Autoantibody profile certainly plays a predictive role in the development of SRC. Anti-RNA polymerase III has a higher prevalence in diffuse SSc and is strongly associated with SRC. Up to 50% of patients with anti-RNA pol III will go on to develop SRC [14, 42–44]. A study from the EUSTAR registry involving 2800 subjects demonstrated anti-RNA polymerase was independently associated with SRC (odds ratio 5.86, 95% confidence interval 2.6, 13.2) [15]. Renal crisis occurs in 10% of patients with anti-topoisomerase (ATA) antibodies [42] which is also associated with diffuse disease. In comparison, there a very few reported cases of SRC in limited anti-centromere antibody (ACA)–positive disease [45].

Outcomes in SRC depending on presence of anti-RNA polymerase III have been compared in a cohort from the Royal Free Hospital. Patients with anti-RNA polymerase III antibodies were more likely to require dialysis but were also more likely to discontinue dialysis (53% vs 26%, *p* = 0.01) and had better long-term survival (*p* = 0.003) [46].

### Genetic Factors

HLA-DRB1*1407 and HLA-DRB1*1304 were identified as independent risk factors for SRC in a study examining over 1500 patients [47]. There has also been suggestion of an association between anti-RNA polymerase III antibody and endothelin receptor A (EDNRA alleles H323H/C and E335E/A) polymorphism but the functional significance of this is yet to be determined [48].

A recent study exploring protein expression in SSc patients who were anti-RNA polymerase III positive found that there was increased expression of two candidate proteins, GPATCH2L and CTNND2, on biopsy staining in SRC patients compared to normal controls. This may help towards explaining why certain groups of anti-RNA polymerase patients are more susceptible to SRC that others [49].

### Clinical Risk Factors

SRC is recognised in a subset of patients who are yet to evolve to diffuse cutaneous disease. These patients are likely to be in the early years of their disease and often display specific disease features suggestive of diffuse subtype such as tendon friction rub, polyarthritis, swollen hands, and/or carpel tunnel [50] and will go on to develop skin thickening.

Historical studies have established that risk factors for development of SRC include diffuse disease, anaemia, pericardial effusion, and congestive heart failure [51]. Rapid progression of skin thickening was also found to be an independent risk factor for SRC [52] as is anti-RNA polymerase III antibody status, tendon friction rub, large joint contractures, heart enlargement [53], proteinuria, and corticosteroid use [15], as demonstrated in Table 2.

**Table 2:** Odds ratio (OR) and hazard ratio (HR) in cohort studies analysing independent risk factors for development of SRC. *CI*, confidence interval 95%; *DcSSc*, diffuse cutaneous SSc; *LcSSc*, limited cutaneous SSc; DLCO, transfer factor as measured by spirometry

	*p* value	OR	HR	CI	Study
anti-RNA pol III	< 0.001	5.86		[2.6–13.2]	Moinzadeh et al. 2020^a^
Chronic kidney disease	< 0.004	2.5		[1.34–4.6]	
	< 0.001		20.7	[2.2–190.7]	Gordon et al. 2019^b^
Proteinuria	< 0.001		183	[19.1–1750]	
	< 0.001	5.55		[3.4–8.9]	Moinzadeh et al. 2020^a^
DcSSc vs. LcSSc	0.002	2.54		[1.42–4.5]	
DLCO	< 0.001	4.41		[2.01–9.6]	
Glucocorticoid use	0.007	1.93		[1.20–3.1]	
	0.014	3.63		[1.30–10.05]	De Marco et al. 2002^c^
	0.49		1.32	[0.60–2.87]	Butikofer et al. 2020^d^
Hypertension	0.002		2.22	[1.34–3.6]	
	< 0.001	13.1		[4.7–36.6]	Gordon et al. 2019^b^
mRSS > 14		3.08		[1.24–7.61]	Avouac et al. 2016^e^
	0.003	10		[2.21–45.9]	De Marco et al. 2002^c^
ACE inhibitors	0.003		2.07	[1.28–3.36]	Butikofer et al. 2020^d^
Tendon friction rub	0.15		1.7	[0.83–3.48]	
	0.0007		2.33	[1.03–6.19]	Avouac et al. 2016^e^
Large joint contracture	0.008	16.12		[2.07–125.2]	De Marco et al. 2002^c^
Heart involvement	0.048	2.93		[1.01–8.4]	

^a^Moinzadeh et al. [15], ^b^Gordon et al. [76], ^c^De Marco et al. [53], ^d^Butikofer et al. [77], ^e^Avouac et al. [50]

Glucocorticoids, particularly at high dose (> 15 mg/day), have long been associated with development of SRC [51]. It has been suggested that glucocorticoids may directly contribute to SRC by inhibiting prostacyclin production and inducing activity of angiotensin-converting enzyme (ACE) [54]. The patients most likely to be taking steroids are those with early and severe disease, who are also at increased risk of SRC which may confound data when discussing the link between steroids and SRC. Data from the ISRCS showed that every 1 mg of prednisolone a patient was taking prior to onset of SRC increased risk of death by 4% (hazard ratio 1.04, 95% CI 1.02, 1.07, *p* < 0.01) [55]. High-dose steroid use is avoided, particularly in early diffuse disease.

By understanding the risk factors which predispose certain patients to development of SRC, the condition can be rapidly recognised and treatment with ACEi initiated promptly. Prompt treatment improves patient outcome [17].

## Outcomes

Outcomes in SRC remain poor compared to other organ complications of SSc but have improved by 50% since the introduction of ACEi treatment in 1981 [7]. Results from the ISRCS showed 36% mortality and 25% remain on dialysis at 1 year [55]. Permanent dialysis is required in 19–40% of SRC cases [7]. Interestingly in the post-ACE era, there has been no evidence of further improvement in morbidity and mortality, highlighting the need for novel treatments in SRC [7].

Recovery of renal function to achieve dialysis independence can occur up to 2 years after the initial event [7, 11] so decisions about renal transplant are delayed accordingly. Three to 17% of SRC cases will require renal transplant [7]. Considerations prior to transplant include co-existing comorbidities, severity of SSc, and choice of immunosuppression following transplant as calcineurin inhibitors are vasoconstrictors and so can theoretically contribute towards further SRC [56]. Survival for SRC patients is superior in the transplant population (54–91%) compared to those on dialysis (31–56%) with graft survival now similar to that of other ESRF [7].

Recurrence of SRC following renal transplant has been reported from 2 to 9% [7]. Recurrence can also occur in the setting of treated SRC not requiring transplant. SRC has been incorporated into an internationally validated tool for predicting 5-year outcome in diffuse disease due to its impact on overall survival [44].

### The Role of ACEi

Interestingly, it has been found that whilst ACEi reduce mortality as treatment for SRC, prior use of ACEi, or prophylactic use, has been associated with worse long-term outcomes and higher frequency of long-term dialysis after SRC [11, 55, 57]. The most widely accepted explanation for this is that small doses of ACEi are not sufficient to treat SRC but may mask the development of hypertension, an important clinical warning sign, leading to delay in treatment and a less reversible, more chronic process. Indeed, those with normotensive SRC have been shown to have worse long-term outcomes.

The ISRCS found a greater than twofold increased risk of mortality in SRC with prior exposure to ACEi [55]; however, many of the patients on ACEi were for indications other than pure prophylaxis of SRC (two case where prophylaxis was used due to glucocorticoid exposure in a high risk patient) so there is a possibility that the results were confounded by clinical severity [18]. Further studies have not shown conclusive evidence to support the use of ACEi prophylactically.

In practice, cases considered to be particularly high risk such as those undergoing autologous haemopoietic stem cell transplant (AHSCT) due to the high dose of glucocorticoids and IV fluid used are given ACEi therapy a few weeks prior to initiating the AHSTC [58]; however, there is no conclusive evidence that ACEi prophylaxis in this context is beneficial.

## Clinical Presentation

The two hallmark features of SRC are accelerated hypertension and AKI but patients can present with a variety of symptoms including headache, blurred vision, and nonspecific symptoms such as fatigue or dyspnoea. Severe SRC may be evidenced by seizures or symptomatic pericardial involvement at presentation.

It is important to point out that whilst an individual’s blood pressure may fall into the ‘normal’ range, the reading may represent a significant increase in average BP for that individual, e.g. if they normally have an average systolic of 80, an increase to 120 is significant and can represent SRC. It is recognised that around 10% of cases are ‘normotensive’ renal crisis, without a rise in systemic BP.

Cardiac complications of SRC are common and may be complicated by underlying cardiac scleroderma. Most symptoms are a response to the sudden increase in blood pressure caused by activation of the RAA axis and often improve in response to tight blood pressure control with therapeutic agents.

### Diagnosis

The United Kingdom Scleroderma Study Group (UKSSG) has produced diagnostic criteria for SRC [56] (Table 3).

**Table 3:** UKSSG Diagnostic criteria for SRC 2016. Reproduced with permission from Lynch et al. [56]

Diagnostic criteria (essential)
New onset BP > 150/85 mmHg or obtained at least twice over 24 h
Increase ≥ 20 mmHg from usual systolic BP
Acute kidney injury stage 1 or higher:
(> 50% increase in serum creatinine from stable baseline or an absolute increase of 26.5 µmol/L)

Supportive evidence (desirable)
MAHA on blood film, thrombocytopaenia and other biochemical findings consistent with haemolysis
Findings consistent with accelerated hypertension on retinal examination
Microscopic haematuria on urine dipstick and/or red blood cells on urine microscopy
Oliguria or anuria
Renal biopsy with typical features of SRC including onion skin proliferation within the walls of intrarenal arteries and arterioles, fibrinoid necrosis, glomerular shrinkage
Flash pulmonary oedema

### Spectrum of Renal Disease

Some conditions can mimic SRC and indeed are difficult to distinguish both in presentation and response to therapy. There are several cases of TTP reported in SSc patients who were distinguishable by fever and haemorrhagic manifestations [59–61]. There is a possibility the two diseases are variations of the same process and if a diagnosis of TTP is made in SRC, an ACEi should be used regardless of whether TPE is also considered as a treatment for the TTP [62].

As discussed earlier, some groups have suggested that we define SRC cases by pathophysiology, rather than the presenting blood pressure. This can be done by observing the sequence and severity of thrombocytopenia, hypertension, and elevation in creatinine. This allows us to differentiate nd-SRC from SSc-TMA and indeed, other not strictly SSc-related causes of TMA, such as TTP (which would indicate TPE treatment), drug-induced TMA, and DIC. The clinical features, serological findings, and histological features or some important differential diseases, when assessing a patient with AKI, are highlighted in Table 4.

**Table 4:** Summary of the clinical and serological features of SRC, TMA, ANCA-associated glomerular nephritis, and SLE nephritis

	**Serum markers**	**Urinalysis**	**Typical presentation**	**Patient cohort**	**Histopathology**
Scleroderma renal crisis (SRC)	Creatine increased AKI (150% typical) Anaemia (MAHA) Thrombocytopenia Haemolysis Negative DAT	Mild proteinuria (< 2 g/day)and haematuria May be urinary casts	Systolic blood pressure ≥ 140 mm Hg Diastolic blood pressure ≥ 90 mm Hg Acute	Early, diffuse SSc Anti-ARA antibody positive High dose glucocorticoid, tendon friction rub	Glomerular or extraglomerular TMA changes, rarely Juxtaglomerulus hyperplasia. Chronic ‘onion skin’ appearance [22]
Thrombotic microangiopathies (TMA) (causes other than SSC)	AKI MAHA (thrombocytopenia and reticularcytosis, increased LDH and low haptoglobin) ADAMS-13 < 10% activity [78]	Proteinuria haematuria	Fever Haemorrhagic manifestation, confusion, neurological deficit Acute	May occur as part of SRC In adults, often underlying comorbid cause. TTP less likely to see AKI	Intravascular fibrin thrombi with mucoid changes. Intimal proliferation of arterioles. Duplication of GBM with endocapillary hypercellularity
ANCA-associated glomerular nephritis	AKI ANCA (MPO/PR3) positive Eosinophilia Thrombophilia Elevated CRP	Proteinuria (often > 3 g/day) and Haematuria likely significant	Vasculitic rash, pulmonary lesions, peripheral neuropathy, fatigue, weight loss Acute/chronic	> 50 years old MPA, GPA, EGPA, RLV	Pauci immune necrotising glomerulonephritis; focal, crescentic, sclerotic or mixed [79]
SLE-associated glomerular nephritis	AKI possible but not diagnostic dsDNA, anti-Smith Low complement (C3)	Proteinuria (> 4 g/day worst prognosis [80]) in 100%, microscopic haematuria in 80% [81]	Known lupus or new features of disease. Possible nephrotic syndrome, 30% hypertension Acute/chronic	Early disease, increased risk in black patients, male patients	Well-defined grade I–V [81]. Glomerular deposits with positive immunofluorescence. TMA in up to 25% [80]

*AKI* acute kidney injury, *DAT* direct antiglobulin test, *MAHA* microangiopathic haemolytic anaemia, *LDH* lactate dehydrogenase, *TTP* thrombotic thrombocytopenic purpura, *GBM* glomerular basement membrane, *ANCA* anti-neutrophil cytoplasm antibodies, *MPO* myeloperoxidase antibody, *PR3* anti-proetinase-3 antibodies, *MPA* microscopic polyangiitis, *GPA* granulomatosis with polyangiitis, *EGPA* eosinophilic granulomatosis with polyangiitis, *RLV* renal limited vasculitis, *SLE* systemic lupus erythematosus, *dsDNA* double-stranded DNA antibody

### Laboratory Findings

Patients typically present with around 150% increase in creatinine from their baseline [18]. The creatine value can continue to rise despite rapid correction of the blood pressure.

Urine dipstick can reveal haematuria and proteinuria which is normally mild (< 2 g/day). Casts may be present, and these findings are not specific to SRC and can be observed in other hypertensive disease.

MAHA is found in approximately 50% of SRC [18] and is an indicator of TMA. Lab findings along with anaemia include thrombocytopenia and reticulocytosis. Thrombocytopenia is often marked, and the recovery of platelets is often the first sign of response to therapy and can occur even if creatinine continues to rise. Table two summarises the different characteristics of SRC and other observed causes of AKI.

Biomarkers as predictors for outcome have been explored. A retrospective study looking at 19 SRC patients found NTproBNP levels > 360 pg/l were highly correlated with patients requiring dialysis [63].

A recent novel finding is that of urinary proteins as candidate markers for renal disease in SSc. Proteins urinary intracellular adhesion molecule (ICAM-1) and urinary monocyte chemoattractant protein (MCP-1) appear to reflect renal involvement better than serum levels [64]. MCP-1 in particular has previously been shown to correlate with SSc skin disease and lung fibrosis and may play a role locally in fibroblast differentiation. At present, the potential biomarkers have been explored in SSc-CKD but may provide insight into acute renal involvement in future prospective studies.

There is ongoing work assessing the role of doppler-measured ‘renal resistive index’ in SSc. The measurement not only appears to be correlated with systemic vasculopathy in SSc, specifically anti-centromere antibody and PAH development [65], but also provides useful information on renal vasculopathy and prediction of mortality [66].

## Management

### Monitoring

Whilst there are no specific preventative measures recognised for SRC, close observation, particularly in high-risk individuals, is key to ensure early detection and treatment. Observation should take the form of regular home recordings of blood pressure (at least twice weekly) with any sustained increase in BP of > 30 mmHg prompting medical attention.

Symptoms such as headache, visual changes, fatigue, or breathlessness should also prompt patients to check blood pressure. This strategy is reliant on patient autonomy so patient education early in the disease course will play a large role in its success. Initiatives such as a patient ‘warning card’ and access to specialist nurses are particularly helpful in this setting [67].

At regular routine clinical assessment, which would be at least 6 monthly as standard of care, urine dipstick and serum urea and creatinine should be monitored, along with blood pressure.

### Pregnancy

As discussed, early stage SSc is the highest risk period for development of SRC and patients are advised to avoid pregnancy during this time [68]. Pregnancy itself does not increase the risk of SRC [69]. Other serious complications of pregnancy such as pre-eclampsia and HELLP (haemolysis, elevated liver enzymes, low platelet) syndrome can mimic SRC and renal biopsy may be required to differentiate the processes. Renal biopsy carries close to the same risk as the general population early in pregnancy but should be avoided in the third trimester unless it will determine management as the risk of complications increases with gestational age [68]. An ACEi should be started immediately if SRC suspected [68, 70]. Captopril is the preferred choice due to lower risk of foetal renal complications. ACEi carries a teratogenic risk to the foetus but in this scenario, this is outweighed by the life-threatening complication of SRC to the mother. In patients with history of SRC planning a pregnancy, it is recommended that ACEi continue during the pregnancy and BP is optimised prior to conceiving. Again, this strategy is not without teratogenic risks to the foetus and this needs to be discussed clearly in pre-pregnancy counselling [68].

### Current Therapies

**Fig. 3:**
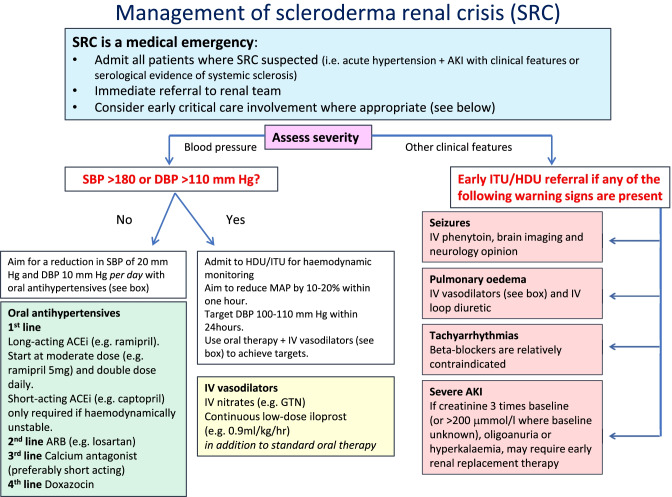
UKSSG guidelines on the diagnosis and management of scleroderma renal crisis Reproduced with permission from Lynch et al. [56]

If evidence of neurological or cardiac compromise is present at onset of SRC, rapid reduction of blood pressure is necessitated. However, if such symptoms are not present, a more gradual reduction in blood pressure can be afforded (10% reduction in systolic BP per day) and confers a better chance of renal recovery [56]. Recommendations for SRC management developed by expert consensus of the UKSSG are shown in Fig. 3.

ACEi should be started as soon as SRC is diagnosed. If the patient is already taking an ACEi, then the dose should be increased. A long-acting ACEi such as ramipril is most used but short-acting agents such as captopril may be suitable in cases of haemodynamic compromise. A long-acting agent is preferable in most cases as it can be easily up-titrated to maximum dose, normally by doubling the dose at 24-h intervals.

Adequate control of blood pressure often takes 3–5 days. It is important to note that renal function can continue to deteriorate despite initiation of ACEi and correction of blood pressure. This should not prompt discontinuation of this important therapy. Additional agents can be added to achieve blood pressure control, including calcium channel blockers, alpha antagonists, and clonidine. Intravenous therapies such as GTN or Iloprost can be used with the latter having the added benefit of discouraging platelet and vascular endothelial activation [56].

ACEi should be continued life-long, even if the patient is dialysis-dependant as an ACEi will improve the chance of the patient subsequently managing to become dialysis-independent [71].

Angiotensin receptor blockers (ARB) can be used if ACEi is contraindicated or not tolerated; however, studies have suggested that ARBs are not clinically equivalent in treatment of SRC [72]. Importantly, ARBs do not inhibit degradation of bradykinin, an agent which is needed in SRC due to its vasodilatory effects. Dual-agent therapy is associated with higher risk of adverse events [18]; therefore, ACEi alone is preferable.

Beta blockers should not be used in SRC due to their negative chronotropic effects on a circulatory system experiencing increased peripheral resistance and may lead to reduction in cardiac output. The use of beta blockers could also exacerbate renal vasospasm ‘renal Raynaud’s’.

It is important to consider differential diagnoses which may have specific treatments that differ to that of SRC. Factors that should prompt consideration of alternative pathologies include a normotensive presentation, significant urinary casts on microscopy, overlap disease phenotype such as SLE or vasculitis, and presentation with fever. In this case, renal biopsy is important and can be sought practically once clotting has normalised and the patient is in a stable condition.

Approximately 60% of SRC will require renal replacement therapy [11] which is often delivered by haemofiltration in the acute setting, moving to haemodialysis or peritoneal dialysis as local resources allow.

TPE is used in settings where related pathologies are suspected such as TTP.

### Immunosuppression

Mycophenolate mofetil (MMF) is an immunosuppressive agent commonly used in SSc and may be initiated following SRC if the patient it not already taking such medication. The rationale behind this being that SRC represents a degree of disease activity which then requires immunosuppression. Rat models of ischaemia and reperfusion to the kidney showed exaggerated production of reactive oxygen species (ROS) leading to cell necrosis [73]. Immunostaining following administration of mycophenolate demonstrated a reduction in interleukin 6(IL-6) and inducible nitric oxygen synthase (iNOS) resulting in restored renal cortical oxygenation. Whether this translates to MMF treatment in SRC has not been proven, a recent retrospective cohort study database did not find any significant association between MMF use and SRC [74].

## Conclusion

SRC is a well-documented complication of SSc but is rare and can present with a variety of symptoms so establishing a core classification criterion is going to be invaluable to future research. Whilst pathological features of SRC are recognised, they are not specific to SRC and there is still much to be understood about why certain predisposed individuals progress to SRC. There could be a role for endothelin and overactivation of the complement pathways, both treatable targets awaiting ongoing trial evidence. The development of sensitive biomarkers for renal disease in SSc may provide further insight into the pathogenesis of SRC whilst also providing tools for early detection and possibly prognosis in SRC. Early, specialist treatment and collaboration between rheumatology and renal physicians will enhance patient outcome. Advances in dialysis delivery and post-transplant management now mean that for those who do go on to require these treatments, the prognosis has improved.
